# Validity and Reproducibility of a Self-administered Food Frequency Questionnaire in the JPHC Study Cohort II: Study Design, Participant Profile and Results in Comparison with Cohort I

**DOI:** 10.2188/jea.13.1sup_134

**Published:** 2007-11-30

**Authors:** Junko Ishihara, Tomotaka Sobue, Seiichiro Yamamoto, Itsuro Yoshimi, Satoshi Sasaki, Minatsu Kobayashi, Tosei Takahashi, Yoji Iitoi, Masayuki Akabane, Shoichiro Tsugane

**Affiliations:** 1 Cancer Information and Epidemiology Division, National Cancer Center Research Institute.; 2 Epidemiology and Biostatistics Division, National Cancer Center Research Institute East.; 3 Department of Nutrition, Tokyo University of Agriculture.

**Keywords:** FFQ, validity, reproducibility, external population

## Abstract

The objective of this study was to evaluate the validity and reproducibility of a self-administered food frequency questionnaire (FFQ) to estimate nutrient and food intake in the subjects of the Japan Public Health Center-based prospective Study on Cancer and Cardiovascular Diseases (JPHC Study Cohort II). The FFQ was originally developed to estimate intake in the JPHC Study Cohort I. A total of 392 subjects were recruited from the entire cohort participants in the 6 areas of Cohort II on a voluntary basis. The subjects completed the FFQ used for the 5-year follow-up survey twice at approximately a 1-year interval. Seven-day dietary records (DR) and blood samples were collected 4 times at 3-month intervals over a year. Daily nutrient and food intakes from FFQ and DR were estimated. The Spearman correlation coefficients for estimated intakes were calculated between FFQ and DR for validity, and between 2 identical FFQs for reproducibility. Correlation coefficients for the validity ranged from 0.09 to 0.82 among various nutrients and food groups. The correlation coefficients for most of the nutrients and food groups were improved to a level comparable to that of Cohort I by energy-adjustment. Correlation coefficients for reproducibility ranged from 0.42 to 0.82, similar to those of Cohort I.

The Japan Public Health Center-based prospective Study on Cancer and Cardiovascular Diseases (JPHC Study) is a large-scale prospective follow-up study in population- and health-checkup-based cohorts. The Cohort I part of the JPHC Study began in 1990 in 5 PHC areas, and the Cohort II part started in 1993 in 6 public health center (PHC) areas. The aim of the study is to investigate the risk and preventive factors including dietary factors for cancer, cardiovascular disease and other chronic diseases.^1^

A self-administered semi-quantitative food frequency questionnaire (FFQ) had been developed to assess dietary intake based on data of 3-day weighed food records collected from random sample in Cohort I areas to be used to estimate dietary intake in the 5-year follow-up survey among Cohort I participants in 1995.^2^ A validation study for estimated nutrient and food intake from FFQ had been conducted among the subsample of 4 areas of Cohort I.^3^ The same FFQ was used for the 5-year follow-up survey in Cohort II in 1998. To examine the validity of the FFQ in the external population, a validation study was conducted among the subsample of 6 areas in Cohort II. The objective of this study was to evaluate validity and reproducibility of the FFQ to estimate nutrient and food intakes in the JPHC Cohort II.

## METHODS

### Study Subjects

**Figure 1.**
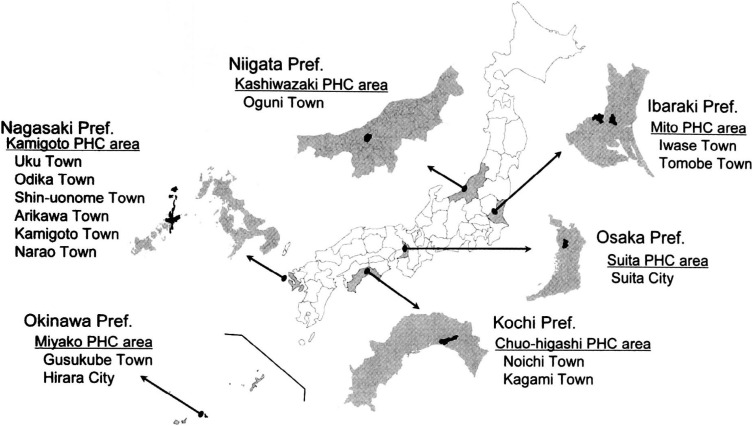
Location map for six areas of JPHC Study Cohort II.

The subjects of the validation study were a subsample of the participants in the JPHC Study Cohort II. The location of the study area is shown in Figure 1, This study was designed according to the study design of the JPHC Cohort I validation study for the FFQ.^3^ The detailed study design and participants of the entire cohort were reported elsewhere.^4^ A total of 392 subjects (196 married couples, 60 from Iwase and Tomobe Towns, Mito PHC area; 60 from Oguni Town, Kashiwazaki PHC area; 76 from Noichi and Kagami Towns, Chuo-higashi PHC area; 66 from Shin-uonome, Arikawa and Kamigoto Towns, Kamigoto PHC area; 66 from Gusukube Town and Hirara City, Miyako PHC area; and 64 from Suita City, Suita PHC area) were recruited on a voluntary basis. Subjects were without any diet restriction, and all of them provided written informed consent.

Although our attempt was to select the subjects from as many cohort participants as possible, some spouses (5 from Kamigoto, 2 from Miyako) were not in the age range of Cohort II participants because we recruited married couples as a unit for the subjects. From Suita PHC area, 30 subjects that included 1 married couple were not cohort participants because the cohort from the Suita PHC area was a random sample not covering the entire population. Among the 30 subjects in Suita, only one was not within the age-range of Cohort II participants (40 to 69 years old at the baseline year).

For the analysis in this report we excluded 7 non-cohort participants from the Kamigoto and Miyako PHC areas. Because 29 non-cohort participants from the Suita PHC area were possible candidates for the random sample, we excluded only one subject whose age was not within the range of Cohort II participants. For the validity analysis, we also excluded 34 subjects who did not complete the DR for 28 days and/or the second FFQ (FFQ_V). For the reproducibility analysis, we excluded 61 subjects without the first FFQ (FFQ_R) from subjects in the validity analysis (Table 1, 2).

**Table 1.** Male participants in validation study

	Mito PHC area	Kashiwazaki PHC area	Chuo-higashi PHC area	Kamigoto PHC area	Miyako PHC area	Suita PHC area	6 PHC areas
Initial registration	30	30	38	33	33	32	196

Questionnaire distributed (FFQ)							
FFQ_B1 (FFQ00)^1^	30	30	38	32	33	32	195
FFQ_B2 (FFQ00)^2^	30	0	0	0	0	0	30
FFQ_R (FFQ05)^3^	30	30	0	31	33	32	156
FFQ_V (FFQ05)^4^	30	30	38	33	31	31	193

Dietary Records (DR)							
DR1	30	30	37	31	33	32	193
DR2	30	30	37	31	33	30	191
DR3	30	30	36	30	33	29	188
DR4	30	30	32	29	33	29	183
28 d completed^5^	29	30	31	28	32	28	178

Blood provided (BL)							
BL1	30	30	33	31	33	32	189
BL2	30	30	27	30	33	30	180
BL3	30	29	23	29	33	29	173
BL4	30	30	31	29	33	30	183
All seasons^6^	30	29	23	27	33	29	171

^1^Baseline questionnaire administered at the beginning of the study. ^2^Second baseline questionnaire. (Only in Mito)

^3^5-year follow-up survey questionnaire administered at the beginning of the study (FFQ for reproducibility).

^4^Second 5-year follow-up survey questionnaire (FFQ for validity).

^5^Subjects who completed DR for all 28 days during data collection.

^6^Subjects who provided blood sample for each of the four seasons.

**Table 2.** Female participants in validation study

	Mito PHC area	Kashiwazaki PHC area	Chuo-higashi PHC area	Kamigoto PHC area	Miyako PHC area	Suita PHC area	6 PHC areas
Initial registration	30	30	38	33	33	32	196

Questionnaire distributed (FFQ)							
FFQ_B1 (FFQ00)^1^	30	30	38	33	33	32	196
FFQ_B2 (FFQ00)^2^	30	0	0	0	0	0	30
FFQ_R (FFQ05)^3^	30	30	0	33	33	32	158
FFQ_V (FFQ05)^4^	30	30	38	33	31	31	193

Diet Records (DR)							
DR1	30	30	37	33	33	32	195
DR2	30	30	38	33	33	30	194
DR3	30	30	36	32	33	30	191
DR4	30	30	32	31	33	30	186
28 d completed^5^	30	30	30	30	33	29	182

Blood provided (BL)							
BL1	29	30	33	33	33	32	190
BL2	29	30	37	32	33	30	191
BL3	29	29	33	31	33	30	185
BL4	29	30	32	32	33	30	186
All seasons^6^	29	29	30	30	33	30	181

### Data Collection

**Figure 2.**
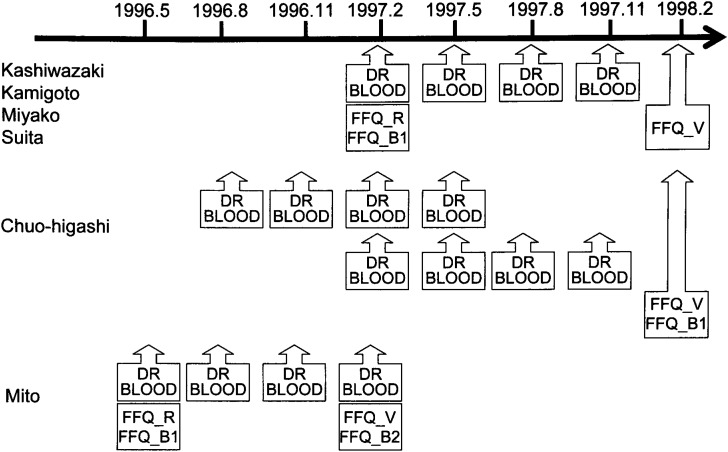
Sequence of data collection for FFQ validation study of JPHC Study Cohort II.

The sequence of data collection was shown in Figure 2.

#### Dietary Records (DR)

The subjects provided 7-day dietary records (DR) in 4 seasons (a total of 28 days): spring (May), summer (August), autumn (November) and winter (February). In Mito the PHC area, the study was launched in the spring of 1996, Half of the subjects from Chuo-higashi (n=32) joined the study in the summer of 1996, and the other half (n=44) in the winter of 1997. In other areas, the study began in winter of 1997.

Weighed DRs were collected over 7 consecutive days in each of the 4 seasons. Dietitians from the PHC, the cities or towns in each area instructed the subjects to weigh all foods and beverages using the measuring spoons, cups and an electronic scale provided, and to record them in a booklet especially designed for the purpose. The subjects gave detailed descriptions of each food, the method of preparation and names of the recipes. The dietitians checked the records at subjects' homes at least once during the survey.

At the end of each season, the dietitians from the PHC reviewed the records in a standardized way, and coded all the foods recorded according to the Standardized Tables of Food Composition, 4th edition,^5^ If codes were not available for certain local foods, the dietitians substituted the food considered to be most similar by asking subjects for details on the food. When ingredients were not obtained for any already prepared recipes, the standard recipes developed by the authors were used.

Nutrient and food calculation was done by the method used in the Cohort I validation study.^6^ The mean daily intake of energy and 16 nutrients was calculated from the records using the Standardized Tables of Food Composition, 4th edition.^5^ For cholesterol, and additional nutrients and compounds such as fatty acids (saturated, monounsaturated, n-3 polyunsaturated, n-6 polyunsaturated)^7^, dietary fiber (water-soluble, -insoluble),^8^ selenium^9^ and carotenoids (alpha-carotene, beta-carotene, lycopene),^10^ the original food composition tables were developed by filling in the missing values for the Japanese composition tables. For isoflavones (daidzein and genistein), the values in the specially developed food composition table for isoflavones in Japanese foods were used.^11^^,^^12^

Additional information about the diet, the standard portions/units for rice and green tea, and brand names for usually used cooking oil, sugar, soy sauce and miso (fermented soybeans) were reported. The frequency of eating out and dietary supplement use for the week was also recorded. Name, age, sex and occupation of all members in the family, self-reported physical activity level, and the number of steps counted by pedometer for one arbitrary day in each season were reported for information on demographic status and physical activity.

#### Food Frequency Questionnaire (FFQ)

Subjects filled in two types of self-administered FFQs used for surveys in the JPHC Study; the FFQ used for the baseline survey (FFQ_B) and the semi-quantitative FFQ used for the 5-year follow-up survey. The latter was repeated twice. The first FFQ (FFQ_R) was to compare with the second FFQ for the evaluation of reproducibility, and the second FFQ (FFQ_V) was to compare with the dietary records for the evaluation of validity. The FFQ_Rs were completed at the same time as the first DR in all but the Chuo-higashi PHC area, where no FFQ_Rs were completed. The FFQ_Vs were completed after a year from the FFQ_R (3 months after completion of last DR) except for the Mito PHC area, where it was nine months after the FFQ_R (same time as last DR). In the Chuo-higashi PHC area, the FFQ was completed in February of 1998.

#### Blood

A total of 22 ml of blood (10 ml in a heparinized tube and the rest in tubes without anticoagulant) was collected by venipuncture from all participants during or after the 7-day in DR each season. All tubes were centrifuged for 10 minutes at 3,500-4,000 r. p. m. within the day of blood collection to obtain plasma, red blood cells and a buffy coat from the heparinized tube, and serum from the other tubes. The plasma, red blood cells, buffy coat and serum were divided into several tubes and frozen in the freezer at each PHC. After the temperature reached -80°C, the tubes, placed in several small boxes, were packed with sufficient dry ice and sent to the National Cancer Center (NCC) by temperature-controlled deliveries. Boxes were immediately put in the freezer at the NCC and stored at -80°C. One of the tubes with serum of each subject was brought to the laboratory of SRL, Inc. (Tokyo, Japan) for the analysis of HDL-cholesterol, total protein, albumin-globulin ratio, total cholesterol, triglyceride, uric acid, gamma-GTP, GOT (AST) and GPT (ALT).

At the time of blood collection, subjects were asked about their health status, medication, and whether they had consumed any foods or beverages within 5 hours. Female subjects were asked about their most recent menstrual period or their age of menopause. Height, weight and blood pressure of the subjects were measured.

### Statistical Analysis

Means and standard deviations of energy, nutrient and food intakes from DRs and FFQ_Vs were calculated by sex. Percent differences between DR and FFQ_V for energy, each nutrient and food were calculated by dividing the difference in mean intake from DR and FFQ by the DR mean, i.e., (FFQ mean-DR mean)IDR mean. Correlation coefficients were calculated using both crude and energy-adjusted values. Nutrient and food group intakes were adjusted for energy using the residual model.

For validity, the correlation coefficients of estimated nutrient and food intakes between DRs and FFQ_Vs were computed. For reproducibility, the correlation coefficient of estimated nutrient and food intakes between FFQ_Rs and FFQ_Vs were computed. Spearman rank correlation coefficients were used for both validity and reproducibility.

## RESULTS

### Participants in Validation Study

For validity analysis, a total of 350 subjects, 174 men (88.8%) and 176 women (89.8%), 59 from Mito (98.3%), 60 from Kashiwazaki (100%), 61 from Chuo-higashi (80.3%), 53 from Kamigoto (80.3%), 60 from Miyako (90.1%) and 57 from Suita PHC areas (89.1%) were included. For the reproducibility analysis, a total of 289 subjects, 143 males and 146 females, were included.

### Profiles of Subjects Completing DRs

The background profile of subjects who completed DRs for 4 seasons and FFQ_V was shown in Table 3. The average age of the subjects was 58 years in men and 55 years in women. The average body mass index (BMI) was 23.7 for men and 23.7 in women. The most frequent occupation indicated was “employee” in men and “housewives” in women, followed by agricultural workers in men and employed workers in women.

**Table 3.** Background characteristics of validation study participants who completed 28-day diet record

	Mito PHC area		Kashiwazaki PHC area		Chuo-higashi PHC area		Kamigoto PHC area		Miyako PHC area		Suita PHC area		6 PHC area	
						
	mean	SD	mean	SD	mean	SD	mean	SD	mean	SD	mean	SD	mean	SD
Male														
n	29		30		31		26		30		28		174	
Age (years old)	57.2	6.5	61.6	6.1	59.5	7.2	55.4	7.6	55.4	8.0	63.9	6.5	58.9	7.6
Height (cm)	165.4	6.2	164.8	4.3	166.3	4.2	162.8	6.1	162.8	6.1	166.3	5.7	164.9	5.5
Weight (kg)	66.0	9.7	63.4	7.3	61.1	8.7	66.0	6.5	68.0	6.6	63.1	7.5	64.6	8.1
BMI^1^	24.1	2.9	23.3	2.3	22.6	2.9	23.9	2.6	25.6	1.7	22.8	2.2	23.7	2.6

Occupation	n	%	n	%	n	%	n	%	n	%	n	%	n	%
n (% of each subject)	29		30		31		26		30		28		174	
Agricultural work	8	27.6	9	30.0	11	35.5	1	3.8	3	10.0	0	0.0	32	18.4
Forestry work	0	0.0	0	0.0	0	0.0	0	0.0	0	0.0	0	0.0	0	0.0
Fishery work	0	0.0	0	0.0	0	0.0	1	3.8	0	0.0	0	0.0	1	0.6
Employee	19	65.5	12	40.0	16	51.6	18	69.2	18	60.0	15	53.6	98	56.3
Self-employed	4	13.8	7	23.3	0	0.0	3	11.5	3	10.0	2	7.1	19	10.9
Professional	4	13.8	1	3.3	0	0.0	2	7.7	3	10.0	1	3.6	11	6.3
Housewife	0	0.0	0	0.0	0	0.0	0	0.0	0	0.0	0	0.0	0	0.0
Unemployed	2	6.9	4	13.3	5	16.1	2	7.7	6	20.0	11	39.3	30	17.2
Other	0	0.0	1	3.3	0	0.0	0	0.0	1	3.3	0	0.0	2	1.1

Female														
n	30		30		30		27		30		29		176	
Age (years old)	54.3	6.4	57.9	6.4	56.6	7.8	53.1	6.4	53.9	7.4	59.4	6.0	55.9	7.1
Height (cm)	152.1	4.7	153.1	5.4	151.1	5.9	154.3	4.8	151.9	4.7	153.7	4.5	152.7	5.1
Weight (kg)	54.6	5.5	55.1	10.0	53.6	7.8	57.2	8.0	56.7	5.5	54.1	8.4	55.2	7.7
BMI^1^	23.6	2.4	23.5	3.9	23.4	3.1	24.0	3.2	24.6	2.7	22.9	3.7	23.7	3.2

Occupation	n	%	n	%	n	%	n	%	n	%	n	%	n	%
n (% of each subjects)	30		30		30		27		30		29		176	
Agricultural work	8	26.7	2	6.7	13	43.3	0	0.0	1	3.3	0	0.0	24	13.6
Forestry work	0	0.0	0	0.0	0	0.0	0	0.0	0	0.0	0	0.0	0	0.0
Fishery work	0	0.0	0	0.0	0	0.0	0	0.0	0	0.0	0	0.0	0	0.0
Employee	9	30.0	8	26.7	6	20.0	6	22.2	17	56.7	8	27.6	54	30.7
Self-employed	1	3.3	3	10.0	0	0.0	4	14.8	0	0.0	1	3.4	9	5.1
Professional	1	3.3	0	0.0	1	3.3	1	3.7	6	20.0	0	0.0	9	5.1
Housewife	12	40.0	19	63.3	10	33.3	17	63.0	11	36.7	20	69.0	89	50.6
Unemployed	2	6.7	1	3.3	2	6.7	1	3.7	2	6.7	1	3.4	9	5.1
Other	2	6.7	1	3.3	2	6.7	2	7.4	1	3.3	1	3.4	9	5.1

^1^Body Mass Index: height (m)/weight (kg)^2^

### Mean Nutrient and Food Intake and FFQ Validity

Daily intakes of energy and nutrients assessed by DR and FFQ and their correlation coefficients were shown in Table 4. The percent difference in nutrient intake between DR and FFQ varied from -33% for selenium to +53% for retinol in men, and from -65% for alcohol to +71% for retinol in women. The correlation coefficients of the crude values varied from 0.17 for polyunsaturated fatty acid (PUFA) to 0.82 for alcohol in men, and 0.21 for n-3 PUFA to 0.52 for alpha-carotene in women. Medians were 0.37 in men and 0.40 in women. After the energy adjustment, the correlation coefficients varied from 0.26 for selenium to 0.65 for calcium in men, and 0.18 for selenium to 0.64 for calcium in women. Medians were 0.49 in men and 0.45 in women.

**Table 4.** Nutrient intakes calculated by DR for 28 days and FFQ_V used for 5-year survey of JPHC Study Cohort II and their correlations

Sex	DR		FFQ_V		% difference^1^	Spearman Correlation	
			
Nutrient	Mean	SD	Mean	SD		Crude	Energy-adjusted^2^
Male (n=174)							
Energy (kcal/day)	2220	348	2132	649	-4	0.34	
Protein (g/day)	89.6	15.4	78.0	30.1	-13	0.29	0.30
Total fat (g/day)	54.9	11.1	60.4	25.6	10	0.26	0.57
Total fatty acid (g/day)	48.0	10.0	54.1	23.1	13	0.28	0.59
SPA^3^ (g/day)	14.8	3.5	18.0	8.7	22	0.42	0.62
MUFA^4^ (g/day)	17.8	3.9	22.8	10.2	28	0.26	0.55
PUFA^5^ (g/day)	15.0	3.1	13.3	5.5	-11	0.17	0.44
n-3 PUFA^5^ (g/day)	3.1	0.8	3.4	1.7	10	0.20	0.30
n-6 PUFA^5^ (g/day)	11.8	2.6	9.9	4.0	-16	0.20	0.49
Carbohydrate (g/day)	297.9	54.0	274.4	84.7	-8	0.40	0.59
Alcohol (g/day)	24.2	24.8	24.4	26.9	1	0.82	0.60
Calcium (mg/day)	646	185	628	374	-3	0.53	0.65
Phosphorus (mg/day)	1348	231	1259	478	-7	0.39	0.49
Iron (mg/day)	12.6	2.4	10.3	4.2	-18	0.27	0.54
Sodium (mg/day)	4681	1183	4739	2406	1	0.29	0.42
Potassium (mg/day)	3238	661	2985	1329	-8	0.33	0.49
Retinol (mg/day)	336	354	515	390	53	0.37	0.35
Carotene (mg/day)	3227	1342	3456	2888	7	0.43	0.47
a-carotene (mg/day)	411	238	520	544	27	0.47	0.50
b-carotene (mg/day)	2583	1120	2712	2303	5	0.40	0.45
Lycopene (mg/day)	3827	2688	2814	3556	-26	0.32	0.29
Vitamin B_1_ (mg/day)	1.3	0.3	1.2	0.5	-10	0.22	0.28
Vitamin B_2_ (mg/day)	1.6	0.3	1.6	0.7	2	0.41	0.55
Niacin (mg/day)	22.0	5.2	18.4	7.9	-16	0.34	0.33
Vitamin C (mg/day)	140	46	159	102	13	0.38	0.46
Cholesterol (mg/day)	393	106	323	184	-18	0.44	0.47
Selenium (*µ*g/day)	181	49	121	56	-33	0.31	0.26
Total dietary fiber (g/day)	15.1	3.5	14.0	6.8	-8	0.41	0.57
Water-soluble fiber (g/day)	2.2	0.6	2.3	1.5	7	0.44	0.54
Water-insoluble fiber (g/day)	10.4	2.5	9.7	4.7	-7	0.39	0.56
Daidzein (mg/day)	14.0	6.3	14.8	9.2	6	0.42	0.49
Genistein (mg/day)	23.2	10.1	24.8	15.5	7	0.41	0.48
Median^6^						0.37	0.49

Female (n=176)							
Energy (kcal/day)	1723	253	1813	639	5	0.22	
Protein (g/day)	72.8	11.1	72.9	32.0	0	0.35	0.31
Total fat (g/day)	48.2	9.5	58.8	28.1	22	0.31	0.40
Total fatty acid (g/day)	42.3	8.7	52.8	25.5	25	0.32	0.42
SFA^3^ (g/day)	13.6	3.4	17.7	9.8	31	0.42	0.51
MUFA^4^ (g/day)	15.4	3.4	21.9	11.0	42	0.31	0.37
PUFA^5^ (g/day)	12.9	2.5	13.1	5.8	2	0.23	0.33
n-3 PUFA^5^ (g/day)	2.5	0.5	3.3	1.6	29	0.21	0.19
n-6 PUFA^5^ (g/day)	10.2	2.2	9.8	4.3	-4	0.27	0.42
Carbohydrate (g/day)	247.0	44.1	247.7	78.9	0	0.24	0.39
Alcohol (g/day)	3	4	1	3	-65	0.47	0.58
Calcium (mg/day)	638	177	660	380	3	0.50	0.64
Phosphorus (mg/day)	1125	182	1189	492	6	0.41	0.54
Iron (mg/day)	11.1	2.0	10.5	4.9	-6	0.39	0.51
Sodium (mg/day)	4019	964	4350	2058	8	0.39	0.45
Potassium (mg/day)	2945	531	3068	1486	4	0.40	0.49
Retinol (mg/day)	287	285	490	534	71	0.44	0.47
Carotene (mg/day)	3069	1159	3855	3270	26	0.47	0.49
a-carotene (mg/day)	380	217	566	559	49	0.52	0.52
b-carotene (mg/day)	2469	971	3067	2689	24	0.47	0.47
Lycopene (mg/day)	4006	2539	2869	2947	-28	0.35	0.37
Vitamin B_1_ (mg/day)	1.1	0.2	1.1	0.5	5	0.33	0.32
Vitamin B_2_ (mg/day)	1.4	0.3	1.6	0.8	14	0.46	0.55
Niacin (mg/day)	16.5	2.9	16.4	8.5	0	0.22	0.22
Vitamin C (mg/day)	144	38	188	121	30	0.42	0.44
Cholesterol (mg/day)	320	87	290	171	-9	0.49	0.47
Selenium (*µ*g/day)	140	34	109	58	-22	0.28	0.18
Total dietary fiber (g/day)	14.4	3.0	15.3	7.8	6	0.42	0.49
Water-soluble fiber (g/day)	2.1	0.5	2.7	1.6	28	0.42	0.46
Water-insoluble fiber (g/day)	9.7	2.1	10.6	5.5	9	0.44	0.50
Daidzein (mg/day)	13.1	5.8	15.9	10.4	21	0.41	0.44
Genistein (mg/day)	21.6	9.4	26.9	17.5	24	0.42	0.45
Median^6^						0.40	0.45

^1^(FFQ mean - DR mean)/DR mean. ^2^Nutrient intakes were adjusted for energy intake by residual model.

^3^Saturated fatty acid ^4^Monounsaturated fatty acid ^5^Polyunsaturated fatty acid.

^6^Median of correlation coefficients for crude intakes of energy and 31 nutrients, and for energy-adjusted intake of 31 nutrients.

Daily food intakes by food groups assessed by DR and FFQ and their correlation coefficients were shown in Table 5. The percent difference between DR and FFQ varied from -78% for seasoning and spices to +48% for non-alcoholic beverages in men, and from -75% to +60% for the same food groups in women. The correlation coefficients of the crude values varied from 0.10 for fungi and algae to 0.77 for alcoholic beverages in men, and 0.09 for fungi to 0.64 for milk and dairy products in women. Medians were 0.35 in men and 0.30 in women. After the energy adjustment, the correlation coefficients varied from 0.11 for algae to 0.69 for milk and dairy products in men, and 0.12 for fungi to 0.65 for pickled vegetables in women. Medians were 0.41 in men and 0.30 in women.

**Table 5.** Food intakes calculated by DR for 28 days and FFQ2 used for 5-year survey of JPHC Study Cohort II and their correlations

Sex	DR		FFQ_V		% difference^1^	Spearman Correlation	
			
Food groups	Mean	SD	Mean	SD		Crude	Energy-adjusted^2^
Male (n=174)							
Cereals	547	131	327	123	-40	0.33	0.33
Potatoes and starches	72.3	25.8	28.8	25.7	-60	0.19	0.28
Confectioneries	66.8	30.7	19.8	28.8	-70	0.30	0.24
Fats and oils	11.7	3.7	13.2	7.0	13	0.14	0.26
Nuts and seeds	8.9	6.8	2.2	3.8	-75	0.30	0.21
Pulses	92.7	34.0	77.2	47.1	-17	0.40	0.52
Fish and shellfish	135	48	97	65	-28	0.29	0.27
Meats	80.1	26.7	62.3	44.9	-22	0.37	0.48
Eggs	50.5	14.6	31.7	29.6	-37	0.47	0.47
Milk and dairy products	161	96	225	275	40	0.71	0.69
Vegetables	320	103	247	178	-23	0.35	0.44
Green & yellow	120	50	74	66	-38	0.35	0.41
Pickled^3^	33.1	23.8	25.5	33.4	-23	0.56	0.56
Fruits	150	72	190	167	27	0.50	0.55
Fungi	21.7	11.4	11.9	10.1	-45	0.10	0.15
Algae	15.3	9.5	12.2	11.7	-20	0.10	0.11
Alcoholic beverage	366	300	282	327	-23	0.77	0.45
Non-alcoholic beverage	542	265	803	425	48	0.44	0.46
Seasonings and spices	38.1	10.6	8.5	5.5	-78	0.15	0.22
Median^4^						0.35	0.41

Female (n=176)							
Cereals	384	89	269	84	-30	0.26	0.22
Potatoes and starches	70.7	23.2	35.3	29.2	-50	0.29	0.30
Confectioneries	70.1	30.0	26.9	30.7	-62	0.25	0.26
Fats and oils	9.8	3.1	12.8	7.0	31	0.16	0.28
Nuts and seeds	8.3	6.3	1.8	2.9	-79	0.12	0.17
Pulses	83.l	25.1	80.5	53.7	-3	0.43	0.54
Fish and shellfish	99	25	89	61	-10	0.29	0.23
Meats	63.2	20.0	52.8	47.0	-16	0.45	0.44
Eggs	43.5	12.9	27.2	24.2	-37	0.50	0.45
Milk and dairy products	192	91	247	262	29	0.64	0.64
Vegetables	294	86	265	184	-10	0.43	0.47
Green & yellow	118	46	85	77	-28	0.35	0.37
Pickled^3^	28.6	17.5	27.8	35.6	-3	0.63	0.65
Fruits	176	62	242	216	38	0.30	0.29
Fungi	20.2	9.7	14.3	12.4	-30	0.09	0.12
Algae	14.7	9.1	11.8	10.1	-20	0.19	0.18
Alcoholic beverage	59	91	20	74	-65	0.48	0.49
Non-alcoholic beverage	527	226	844	440	60	0.42	0.41
Seasonings and spices	33.8	10.0	8.5	5.5	-75	0.14	0.14
Median^4^						0.30	0.30

^1^(FFQ mean - DR mean)/DR mean. ^2^Food intakes were adjusted for energy intake by residual model.

^3^Pickled plum (umeboshi) was included in pickled vegetables, and not in total vegetables but rather in fruits.

^4^Median of correlation coefficients for 19 food groups.

### Reproducibility

Reproducibility of nutrient intake estimated by 2 identical FFQs (FFQ_R and FFQ_V) was shown in Table 6. The correlation coefficient of the crude values varied from 0.49 for lycopene to 0.82 for alcohol in men, and from 0.54 for carbohydrate and genistein to 0.70 for alcohol in women. Medians were 0.61 in men and 0.63 in women. After the energy adjustment, the correlation coefficient varied from 0.46 for alpha-carotene and lycopene to 0.77 for alcohol in men, and from 0.33 for MUFA to 0.72 for alcohol in women. Medians were 0.56 in men and 0.51 in women. Reproducibility was highest for alcohol intake among all the nutrients.

**Table 6.** Spearman rank correlation coefficients between nutrient intakes assessed with two FFQs one year apart (JPHC Study Cohort II)

Nutrients	Male (n=143)		Female (n=146)	
	
	Crude	Energy-adjusted^1^	Crude	Energy-adjusted^1^
Energy	0.63		0.60	
Protein	0.59	0.57	0.68	0.54
Total fat	0.57	0.57	0.62	0.38
Total fatty acid	0.56	0.57	0.62	0.38
SFA^2^	0.56	0.61	0.65	0.53
MUFA^3^	0.56	0.54	0.60	0.33
PUFA^4^	0.62	0.53	0.63	0.35
n-3 PUFA^4^	0.64	0.54	0.64	0.35
n-6 PUFA^4^	0.61	0.57	0.63	0.41
Carbohydrate	0.61	0.55	0.54	0.41
Alcohol	0.82	0.77	0.70	0.72
Calcium	0.69	0.70	0.68	0.61
Phosphorus	0.61	0.67	0.68	0.62
Iron	0.67	0.66	0.68	0.51
Sodium	0.59	0.56	0.69	0.67
Potassium	0.66	0.63	0.65	0.58
Retinol	0.66	0.68	0.66	0.64
Carotene	0.62	0.56	0.68	0.53
a-carotene	0.52	0.46	0.62	0.49
b-carotene	0.60	0.54	0.68	0.52
Lycopene	0.49	0.46	0.62	0.57
Vitamin B_1_	0.56	0.47	0.59	0.45
Vitamin B_2_	0.69	0.66	0.62	0.51
Niacin	0.66	0.39	0.63	0.37
Vitamin C	0.63	0.58	0.59	0.48
Cholesterol	0.59	0.55	0.67	0.57
Vitamin B_6_	0.66	0.54	0.62	0.47
Vitamin B_12_	0.60	0.52	0.57	0.45
Folate	0.64	0.62	0.60	0.51
Selenium	0.57	0.50	0.61	0.43
Total dietary fiber	0.65	0.66	0.63	0.61
Water-soluble fiber	0.64	0.62	0.63	0.58
Water-insoluble fiber	0.63	0.64	0.64	0.60
Daidzein	0.61	0.53	0.55	0.43
Genistein	0.60	0.51	0.54	0.41
Median^5^	0.61	0.56	0.63	0.51

^1^Nutrient intakes were adjusted for energy intake by residual model.

^2^Saturated fatty acid ^3^Monounsaturated fatty acid ^4^Polyunsaturated fatty acid.

^5^Median of correlation coefficients for crude intakes of energy and 31 nutrients, and for energy-adjusted intake of 31 nutrients.

Reproducibility of food intakes estimated by 2 identical FFQs (FFQ_R and FFQ_V) were shown in Table 7. The correlation coefficient of the crude values varied from 0.44 for nuts and seeds to 0.78 for alcoholic beverages in men, and from 0.42 for cereals to 0.74 for milk and dairy products in women. After the energy adjustment, the correlation coefficient varied from 0.38 for algae to 0.70 for alcoholic beverages in men, and 0.40 for fish and shellfish and algae to 0.80 for alcoholic beverages in women.

**Table 7.** Spearman rank correlation coefficients between food intakes assessed with two FFQs one year apart (JPHC Study Cohort II)

Food groups	Male (n=143)		Female (n=146)	
	
	Crude	Energy-adjusted^1^	Crude	Energy-adjusted^1^
Cereals	0.56	0.40	0.42	0.47
Potatoes and starches	0.53	0.52	0.60	0.55
Confectioneries	0.71	0.63	0.54	0.51
Fats and oils	0.57	0.54	0.56	0.48
Nuts and seeds	0.44	0.42	0.53	0.50
Pulses	0.64	0.57	0.53	0.44
Fish and shellfish	0.58	0.46	0.62	0.40
Meats	0.59	0.52	0.58	0.41
Eggs	0.59	0.50	0.64	0.53
Milk and dairy products	0.68	0.69	0.74	0.77
Vegetables	0.63	0.56	0.65	0.59
Green & yellow	0.62	0.59	0.63	0.50
Pickled^2^	0.67	0.67	0.72	0.73
Fruits	0.60	0.57	0.59	0.54
Fungi	0.50	0.49	0.52	0.49
Algae	0.49	0.38	0.52	0.40
Alcoholic beverage	0.78	0.70	0.70	0.80
Non-alcoholic beverage	0.56	0.48	0.58	0.52
Seasonings and spices	0.64	0.56	0.54	0.41
Median^3^	0.59	0.54	0.58	0.50

^1^Food intakes were adjusted for energy intake by residual model.

^2^Pickled plum (umeboshi) was included in pickled vegetables, and not in total vegetables but rather in fruits.

^3^Median of correlation coefficients for 19 food groups.

## DISCUSSION

In this study, we evaluated the validity and reproducibility of the FFQ in the external population for which the FFQ was originally developed. From 392 subjects, we collected 2 types of FFQ; the FFQ used for the baseline survey (FFQ_B) and two identical FFQs used for the 5-year follow-up survey (FFQ_R and FFQ_V). We also collected 7-day DRs and blood samples from each of the 4 seasons. In this report, we estimated daily nutrient and food intakes from the DR, FFQ_R and FFQ_V. The correlation coefficients of nutrient and food group intakes between DR and FFQ_V were computed to evaluate validity of the FFQ. The correlation coefficients of nutrient intake between FFQ_R and FFQ_V were computed to evaluate reproducibility of the FFQ.

**Figure 3.**
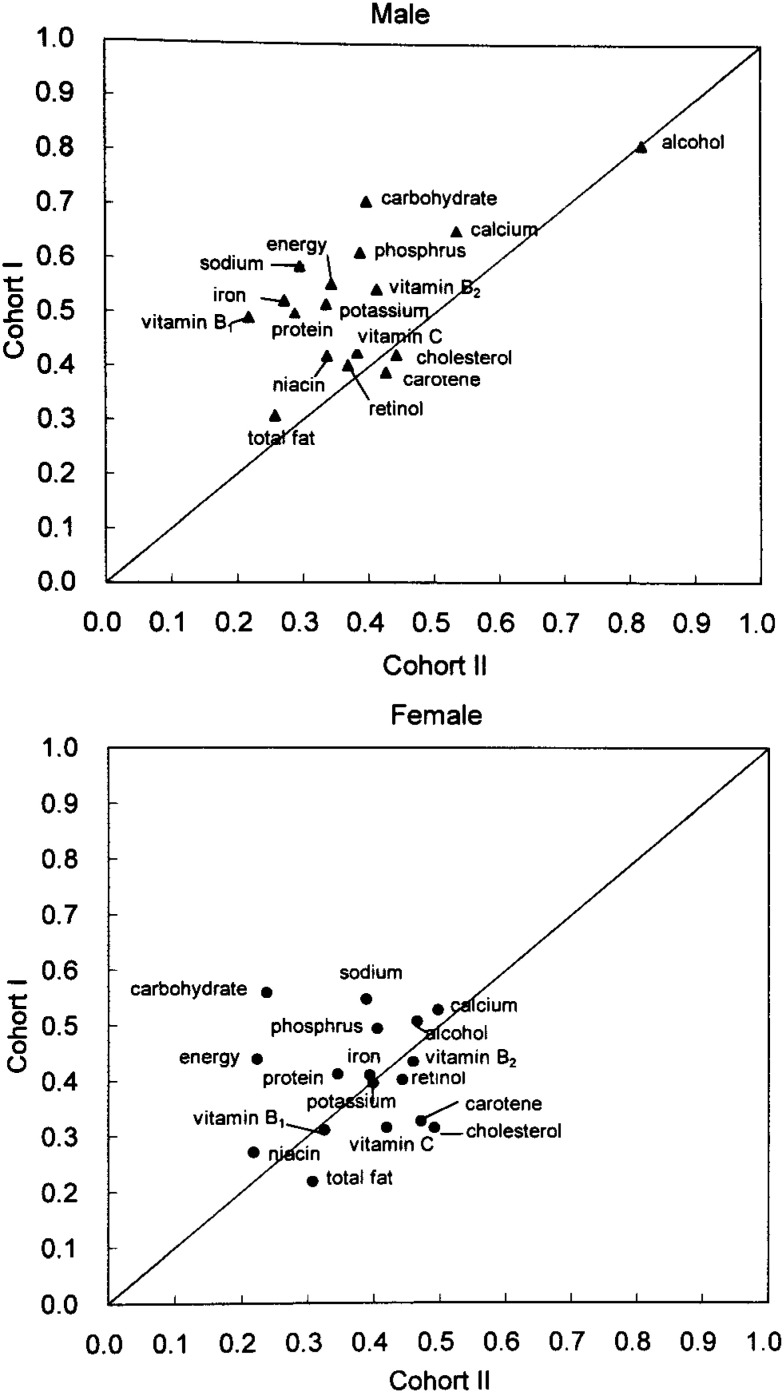
Comparison of correlation coefficients for selected nutrients (crude) between Cohort I and II.

**Figure 4.**
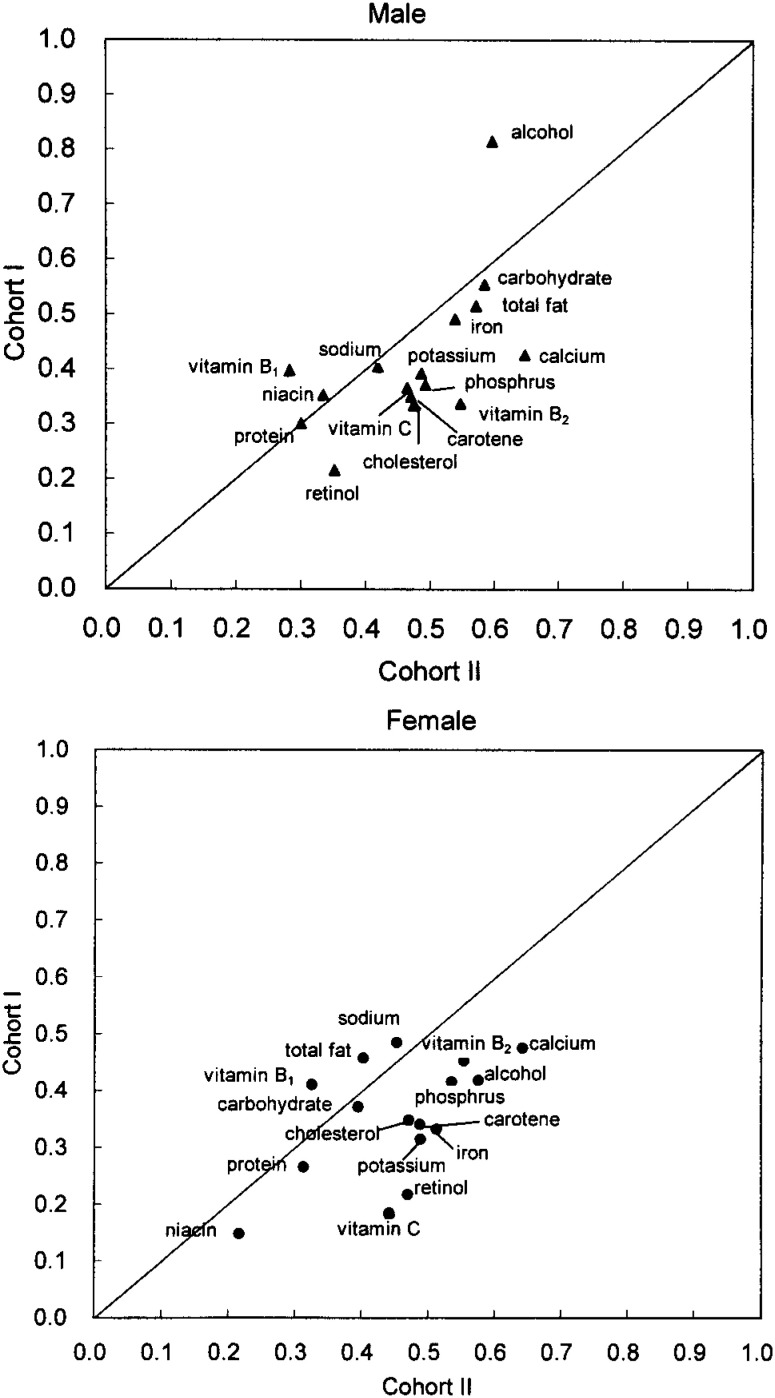
Comparison of correlation coefficients for selected nutrients (energy-adjusted) between Cohort I and II.

The validity of the FFQ to estimate energy and nutrient intake in Cohort II was slightly lower for most of the nutrients compared to Cohort I.^13^ Correlation coefficients for energy and 16 crude nutrient intakes from this study were compared to those from the validation study of Cohort I in Figure 3. Except for alcohol in men, the correlation coefficients for each nutrient in Cohort II did not agree with the corresponding nutrients in Cohort I (r=0.30 for men and 0.11 for women after excluding alcohol). However, the validity for estimated nutrients was improved to a comparable level of Cohort I by energy adjustment, except for alcohol in men (Figure 4). The correlation coefficients became similar for the same nutrients between Cohort I and Cohort II (r=0.61 for men and 0.57 for women after excluding alcohol). We assumed that the error associated with the estimation of dietary intake was offset by adjusting nutrient intake by total energy intake. The alcohol intake in men, however, might not have included the same error as the others because alcohol consumption did not depend on diet in men.

**Figure 5.**
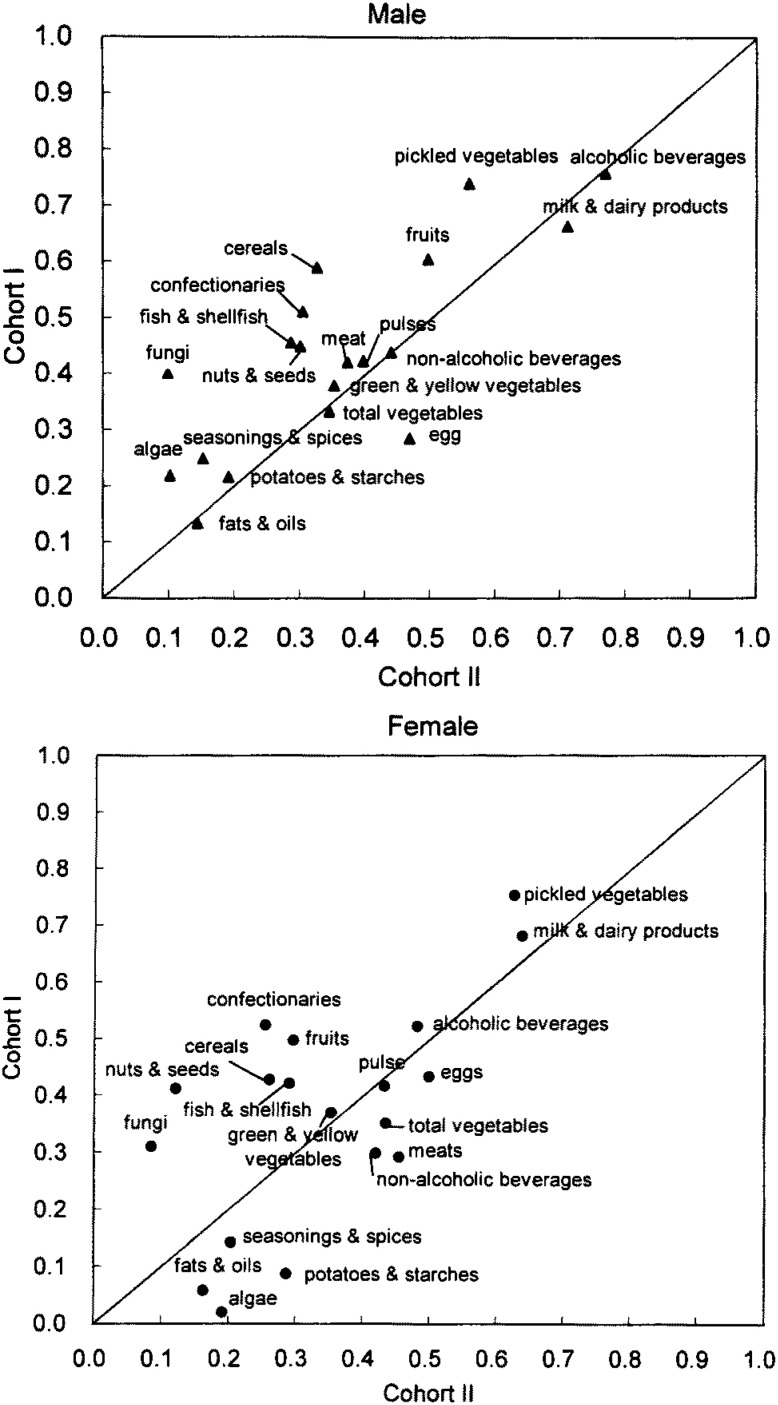
Comparison of correlation coefficients for selected food groups (crude) between Cohort I and II.

**Figure 6.**
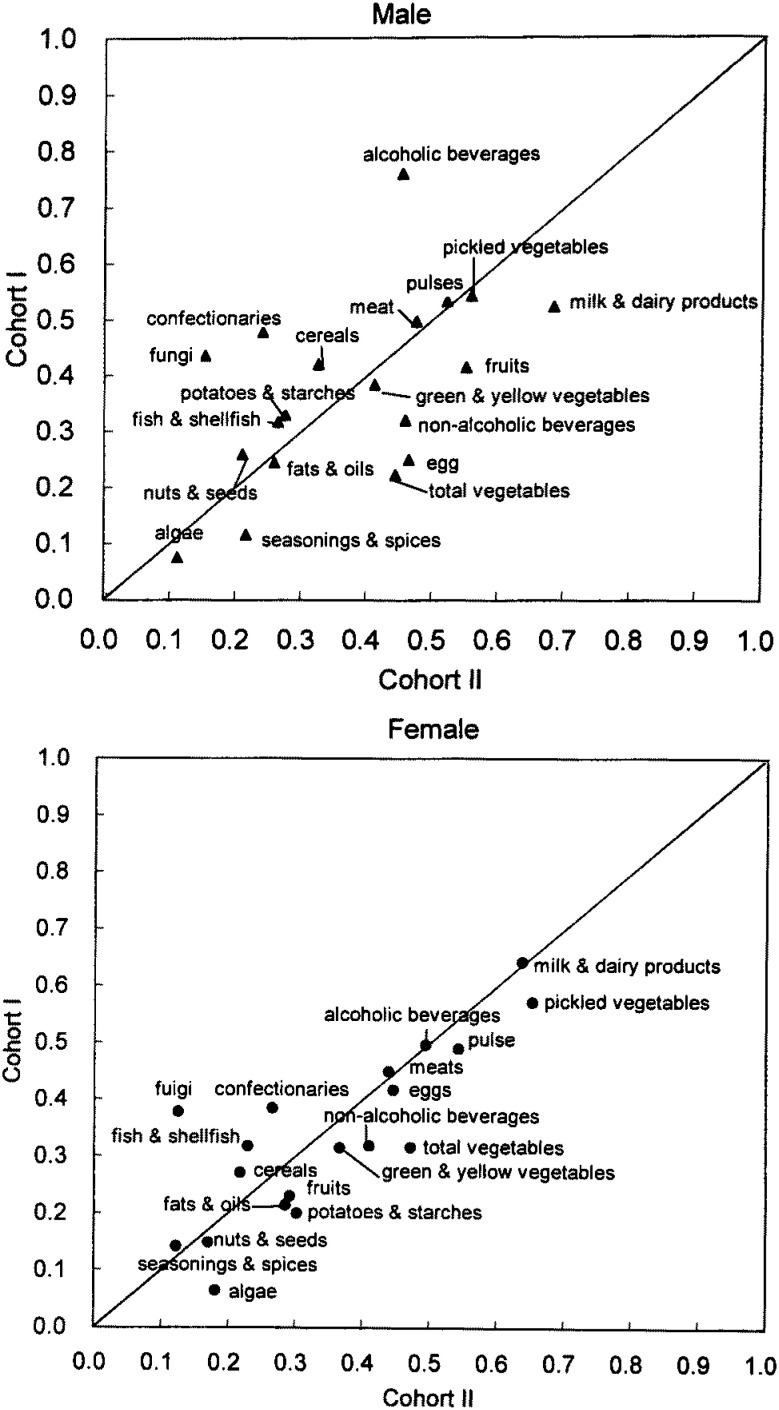
Comparison of correlation coefficients for selected food groups (energy-adjusted) between Cohort I and II.

The validity of the FFQ to estimate food intake in Cohort II was slightly lower than that in the Cohort I for most of the food groups.^14^ Correlation coefficients for food group intakes (crude values) from this study were compared to those from the validation study of Cohort I in Figure 5. Food groups with low correlation coefficients in Cohort I such as potatoes and starches, fats and oils, algae, and seasonings and spices were also low in Cohort II, while food groups with high correlation coefficients in Cohort I such as milk and dairy products, and pickled vegetables were also high in Cohort II. Correlation coefficients for most of the food groups improved after the energy adjustment, especially in men (Figure 6).

Reproducibility of the FFQ to estimate nutrient and food intakes in Cohort II was similar to that in Cohort I (0.41-0.83 in men and 0.52-0.77 in women for crude nutrient intake, 0.42-0.80 in men and 0.45-0.74 in women for crude food intake).^15^ The correlation coefficients were slightly lower after energy adjustment, reflecting the same tendency in Cohort I. We initially speculated that there would be lower reproducibility in Ibaraki because of the FFQ interval of 9 months, against a year in other areas. However, the results of subgroup analysis did not show any difference between Ibaraki and other areas. This might be a result of the design of our FFQs which assess the usual dietary intake over a year by asking subjects their highest seasonal consumption of certain foods in light of the high seasonal variety. The result contradicted the result from a previous study which indicated lower reproducibility when FFQs were repeated in different seasons compared to when repeated at a year's interval.^16^

The reproducibility of our FFQ was comparable to findings in the literature in terms of the dietary intake estimated by the FFQ. Correlation coefficients between repeated measurements for estimating dietary intake are usually on the order of 0.5-0.7.^17^ This level of reproducibility is comparable to that of many biological measurements such as serum cholesterol and blood pressure, which are strong and consistent predictors of disease in epidemiologic studies^17^ Except for lycopene, nuts and seeds, algae in men and cereal in women, the reproducibility of our FFQ for estimating intake of nutrients and food groups was r>0.5, which is sufficiently reasonable for epidemiologic use. This reasonable degree of reproducibility ensures that our FFQ can repeatedly assess the intake of individuals over a year. Given its reasonable validity, we can be sure that our FFQ at one point in time could obtain the usual intake of individuals over the period of a year.

In the present report, we did not discuss the validity of the FFQ in relation to biochemical markers because the ongoing analysis is still in the early stages. Those biochemical indicators, however, are an important resource for comparison of dietary intake when used in conjunction with DR^17^ for further analysis. Biochemical measurements such as blood levels of vitamin C, carotenoid, phospholipid, selenium, folate, vitamin B_6_, vitamin B_12_ and isoflavones have been used for analysis to validate the FFQ in Cohort I.^8^^,^^18^^-^^21^ Analysis for validation and the seasonal variation using these biochemical measurements and dietary data of DR from each of the seasons is currently in progress in Cohort II.

In conclusion, the validity of our FFQ for use with Cohort II was comparable to the validity in Cohort I after the energy adjustment. Reproducibility of the FFQ in Cohort II was similar to the reproducibility in Cohort I. Both validity and reproducibility were at a reasonable level, showing that the FFQ was accurately estimating dietary intake of individuals for epidemiologic use. The results of our study thus indicated the possible use of FFQs in the external population for which it was originally developed.
